# Early leucine programming on protein utilization and mTOR signaling by DNA methylation in zebrafish (*Danio rerio*)

**DOI:** 10.1186/s12986-020-00487-3

**Published:** 2020-08-14

**Authors:** Qiang-Sheng Zhu, Jie Wang, Shan He, Xu-Fang Liang, Shuang Xie, Qian-Qian Xiao

**Affiliations:** 1 College of Fisheries, Chinese Perch Research Center, Huazhong Agricultural University, Wuhan, 430070 China; 2 Innovation Base for Chinese Perch Breeding, Key Lab of Freshwater Animal Breeding, Ministry of Agriculture, Wuhan, 430070 China

**Keywords:** Leucine, Early nutritional programming, mTOR singling pathway, Protein synthesis, Glycolipid metabolism, DNA methylation

## Abstract

**Background:**

Early nutritional programming affects a series of metabolism, growth and development in mammals. Fish also exhibit the developmental plasticity by early nutritional programming. However, little is known about the effect of early amino acid programming on growth and metabolism.

**Methods:**

In the present study, zebrafish (*Danio rerio*) was used as the experimental animal to study whether early leucine stimulation can programmatically affect the mechanistic target of rapamycin (mTOR) signaling pathway, growth and metabolism in the later life, and to undercover the mechanism of epigenetic regulation. Zebrafish larvas at 3 days post hatching (dph) were raised with 1.0% leucine from 3 to 13 dph during the critical developmental stage, then back to normal water for 70 days (83 dph).

**Results:**

The growth performance and crude protein content of zebrafish in the early leucine programming group were increased, and consistent with the activation of the mTOR signaling pathway and the high expression of genes involved in the metabolism of amino acid and glycolipid. Furthermore, we compared the DNA methylation profiles between the control and leucine-stimulated zebrafish, and found that the methylation levels of CG-differentially methylated regions (DMGs) and CHH-DMGs of genes involved in mTOR signaling pathway were different between the two groups. With quantitative PCR analysis, the decreased methylation levels of CG type of Growth factor receptor-bound protein 10 (*Grb10*), eukaryotic translation initiation factor 4E (*eIF4E*) and *mTOR* genes of mTOR signaling pathway in the leucine programming group, might contribute to the enhanced gene expression.

**Conclusions:**

The early leucine programming could improve the protein synthesis and growth, which might be attributed to the methylation of genes in mTOR pathway and the expression of genes involved in protein synthesis and glycolipid metabolism in zebrafish. These results could be beneficial for better understanding of the epigenetic regulatory mechanism of early nutritional programming.

## Introduction

The nutritional programming stimulus exerted at the critical stages of early ontogeny might have persistent consequences on physiological functions in later life stages in mammals [1]. Early nutritional programming is one of the important methods to change the metabolic reaction in later life [2]. Previous studies have been undertaken in fish to determine if the metabolic pathway can be influenced in later life via early nutritional programming [3–6]. At the critical stage of early development of the individual, fish also have significant developmental plasticity by early nutritional programming, as in mammals [7]. Early long-chain n-3 high unsaturated fatty acids programming during the critical developmental stage could have a persistent impact on growth performance and lipid metabolism in later life of Siberian sturgeon (*Acipenser baerii* Brandt) [8]. In rainbow trout (*Oncorhynchus mykiss*), early high-carbohydrate diet stimulation has a lasting impact on the genes and enzymes of carbohydrate digestion, glucose transport and metabolism at juvenile stage [3, 9, 10]. However, little is known about the effects of early amino acid programming on growth and nutritional metabolism.

Leucine plays a vital role in protein synthesis and degradation in mammals [11]. The lack or excess of dietary leucine levels reduces the feed utilization and thus affects growth performance in fish [12–14]. In mammals, dietary leucine supplementation has been verified to stimulate protein synthesis via activating the mTOR pathway [11]. Leucine can activate the mTOR signaling pathway in central nervous system of rainbow trout, thereby regulating the digestion and absorption of nutrients [15]. In rainbow trout hepatocytes, leucine stimulation could regulate the mTOR signaling pathway, lipogenesis and gluconeogenesis [16, 17]. However, the knowledge of effect of early leucine programming on nutritional metabolism through mTOR signaling pathway is still limited.

Epigenetic modification is one of the most likely candidates for working on the nutritional programming. Previous researches have mainly focused on the epigenetic regulation mechanisms of nutritional or metabolic programming [18, 19]. DNA methylation is one of the most intensely studied epigenetic modifications, and plays a vital role in the regulation of biological processes, such as cell differentiation, embryogenesis, genomic imprinting and gene expression [20, 21]. Early methionine programming has been shown to affect the DNA methylation in later life via controlling one-carbon metabolism in mammals [22, 23]. In rainbow trout, the methionine level could be of critical importance in metabolic programming, and modified DNA methylation levels at some specific loci of *bnip3a* and *bnip3lb1* [24]. However, few data are available on epigenetic regulatory mechanisms of early leucine programming in fish.

In the present study, we used the leucine immersion at early stage of development of zebrafish to study whether early leucine stimulation can programmatically affect the mTOR signaling pathway, growth and nutrition metabolism, and to investigate the DNA methylation involved in the early leucine programming. This is the first study to explore the global methylation profile of early amino acid programming in zebrafish. It might provide a theoretical basis for the molecular regulatory mechanisms of early nutritional stimulation on growth and metabolism in later life of animals.

## Materials and methods

### Fish and samples

All zebrafish embryos used in this study were obtained from Institute of Hydrobiology, Chinese Academy of Sciences (Wuhan), and hatched in a 28 °C incubator. The zebrafish larvas were maintained in the circulating water system at 27–28 °C (12 h light: 12 h dark photoperiod). The larvas at mouth opening period were fed with egg yolk twice per day, and then a gradual replacement with brine shrimp occurred from 5 dph, till the larvas were exclusively fed with brine shrimp, which was one of the general foods for zebrafish. The brine shrimp larvas were purchased from Tianjin Fengnian aquaculture Lit. (Tianjin, China), and hatched in a salty water of 16–17 ‰ at 27 ± 1 °C for 24 h. The brine shrimp contains 49.6% of crude protein, 13% of moisture, 3.4% of ash, 5.2% of crude fat. To eliminate the effect of change of diet on gene or protein expression, zebrafish was fed with brine shrimp during the whole experiment period. The zebrafish larvas at 3 dph were randomly assigned into the control group (without programming) and the leucine programming group with three replicates (*n* = 300) for each group. Larvas in the leucine programming group were cultured in the water with 1% leucine (pH = 6.8 ± 0.1) for 10 days from 3 to 13 dph, and then reared in normal water for 70 days from 13 to 83 dph. The immersion treatments were conducted as a similar manner described by the previous study [25]. The control group was cultured in normal water (pH = 7.2 ± 0.1) during the whole experiment period. The zebrafish larvas were sampled at 13 and 83 dph, respectively. The body weight and total length were measured. The experiments were performed in accordance with the “Guidelines for Experimental Animals” of the Ministry of Science and Technology (Beijing, China). The study was approved by the Institutional Animal Care and Use Ethics Committee of Huazhong Agricultural University. All efforts were made to minimize suffering.

The whole fish (13 dph) were sampled for analyzing leucine content in whole-body of fish with the leucine immersion treatment. The whole-body samples were freeze-dried and finely ground using a grinder, and acid hydrolysis was performed. After filtration with a 0.22 μm membrane filter, the analysis of leucine was carried out by using A300 amino acid analyzer (membraPure Bodenheim, Germany) [26], with the experiment parameter: sulfonic acid cation resin chromatography column (4.6 mm × 60.0 mm), column temperature of 57.0 °C, reactor temperature of 130 °C, the flow rate of pump A (elution solution) of 0.40 mL/min, the flow rate of pump B (ninhydrin solution) of 0.35 mL/min, the sample volume of 20 μL, the detection wavelength of 570 nm.

### Body composition analysis

Six zebrafish at 83 dph were randomly selected and stored at − 20 °C, which were used for body composition analysis. The whole-body compositions were determined by the standard methods [27]. The moisture was analyzed by drying at 105 °C for 6 h. By using the Kjeltec system after acid digestion (K8400 Kjeltec Analyzer, Fossana Lyticab, Sweden), the determination of crude protein (N × 6.25) was conducted. The crude lipid was measured by using the ether-extraction with Soxtec System HT (SE-A6, Alvah, China).

### Real-time qPCR analysis

Total RNA was extracted from whole fish (13 dph) and liver tissue (83 dph) using TRIzol reagent (TaKaRa, Japan) and purified for expression analysis of mRNA. Afterwards, total RNA was reverse transcribed via using HiScript II Q RT SuperMix reverse transcriptase (Vazyme, Piscataway, NJ, USA). After complementary DNA (cDNA) synthesis, the expression level of mRNA was detected according to ChamQ SYBR qPCR Master Mix (Vazyme, Piscataway, NJ, USA). Design primers according to Primer5 software, expression levels of mRNA were analyzed by a CFX Maestro real-time detection system (Bio-Rad, USA). Relative gene expression was calculated using the 2^−∆∆Ct^ method [28], each sample was repeated at least three times. Primers of all genes for Real-time qPCR analysis are listed in Table 1.

**Table 1:** Primers of all genes for qRT-PCR are listed

	Sequence 5′-3′	Tm (°C)
*accα*	F: GTGGAAACAAAGTTATTGAGAAGG	55
	R: GTAAGCCCAGCGTCGGA	
*cpt1*	F: ATCAGCACTGTTGAGCGAAG	59
	R: CACTCCCTCCCTACTTATCTCC	
*cs*	F: TTCGCTCGGGCGTATTCT	59
	R: GCTGCTGCCTTCACGGTAT	
*fas*	F: GATGGACGAGTGCTTTACCC	55
	R: ATGGTGGCTCTATGGATGGT	
*got*	F: GCTAAAGGCTTACACCTACTAT	56
	R: GTCAAAGAACACCAGGAGAT	
*gpt*	F: AGAAGACCCTGACGATGGAC	56
	R: GAGGAAGGTGATTGGTTGCT	
*Pfk1*	F: AACGAACTCTTCCAAACTCCTG	55
	R: GACTCCTTCATACGCCTCAAAT	
*mtor*	F: GCCGCTTTGCCAACTATTT	55
	R: TCGTCTGCCTTCATTCCTG	
*leptinA*	F: ATTCCCGCTGACAAACCC	56
	R: GTAACCCAGAAGTGTGGATAGATC	
*leptinB*	F: CCCCGTCACCTCCAACTACCT	59
	R: CAGAGAATGAATGTCTCAGCCACA	
*grb10*	F: TCCGAACCCTTTCCCTGAG	60
	R: CTTCCACAACTTTTCCCACA	
*wdr24*	F: TTACTGAGCGGCAAACCC	57
	R: TGATTCGCAGCATCGTCC	
*eif4e*	F: AGTGATGATGTCTGTGGTGCTG	61
	R: TGTTCTCGTAGTCTGTCGTCC	
*β-actin*	F: CACCTTCCAGCAGATGTGGA	58
	R: AAAAGCCATGCCAATGTTGTC	

### Western blot analysis

The whole fish (13 dph) and liver tissues (83 dph) stored at − 80 °C were solubilized in RIPA lysis buffer. The protein content was determined using BCA protein assay kit (Yeasen, China). The proteins were separated on 10% SDS-PAGE gel, and then transferred onto PVDF membrane. Anti-phospho ribosomal protein S6 kinase 1 (S6K1) (Thr389), anti-S6K1, anti-phospho ribosomal protein S6 (S6) (Ser235/236), anti-S6 and anti-phospho Grb10 (Ser476) were purchased from Cell Signalling Technology (USA), anti-β-actin antibody from Bioss (China), anti-β-tubulin antibody from Zoman Biotechnology (China). Blots were probed by goat anti-rabbit and goat anti-mouse second antibody with IR-Dye 680 or 800cw labeled (Licor, USA) at room temperature for 1 h. The membranes were then visualized using a LiCor Odyssey scanner (Licor, USA) and quantified with ImageJ 1.44 software (National Institute of Health, MD). The phosphorylation level of S6 and S6K1 were normalized according to the loading of proteins by expressing the data as a ratio of phospho-S6 and phospho-S6K1 over S6 and S6K1, respectively. Besides, the phosphorylation level of Grb10 were normalized according to the loading of proteins by expressing the data as a ratio of phospho-Grb10 over β-actin.

### Methylome sequencing

The genomic DNA was extracted from the liver tissue of zebrafish (83 dph) with a DNA extraction kit (Tiangen, China), and the DNA concentration was determined with a multi-function microplate reader (BioTek, USA). The contamination and degradation of genomic DNA was examined with 1% agarose gel electrophoresis. For whole genome bisulfite sequencing (WGBS) technology library constructing, the genomic DNA was fragmented to an average size of approximately 250 bp by sonication using a Bioruptor (Diagenode, Belgium), followed by end repair and adenylation. Ligated DNA was bisulfite converted using the EZ DNA Methylation-Gold kit (ZYMO). Different insert size fragments were excised from the same lane of a 2% TAE agarose gel. Products were purified by using QIAquick Gel Extraction kit (Qiagen) and amplified by PCR. Sequencing was performed using HighSeq4000 platforms. The library construction and sequencing were performed by Beijing Genomics Institute (BGI)-Shenzhen (Shenzhen, China).

After filtering adaptor sequences, contamination and low-quality reads, the clean reads data was mapped to the reference genome of zebrafish (*Danio rerio* assembly GRCz11, https://www.ncbi.nlm.nih.gov/genome/?term=Danio+rerio%5Borgn%5D) by Bisulfite Sequence Mapping Program (BSMAP), and then removed the duplication reads and merged the mapping results according to each library. We calculated the mapping rate and bisulfite conversion rate of each sample. The DMRs between the control group and leucine programming group were identified by comparison of the sample methylomes from two groups using windows that contained at least 5 CpG (CHG or CHH (H = C, T and A)) sites with a 2-fold change in methylation level, and Fisher Test *P* value ≤0.05. Gene Ontology (GO) and Kyoto Encyclopedia of Genes and Genomes (KEGG) enrichment analysis of genes related to DMRs were considered significantly enriched KEGG and GO terms with corrected *P* values of less than 0.05. DNA methylation status of the genes involved in mTOR signaling pathway were displayed by Integrative Genomics Viewer (IGV 2.8.x).

### Statistical analysis

Statistical analyses were performed with SPSS 19.0 software. All data were tested for normality and homogeneity of variances using the Shapiro-Wilk’s test and Levene’s test, respectively. Significant differences were found using one-way analysis of variance (ANOVA), followed by Fisher’s least significant difference post hoc test and Duncan’s multiple range tests, after confirming data normality and homogeneity of variances. Differences were considered be significant if *P* < 0.05.

## Results

### Effects of early leucine programming on growth and body composition

**Fig. 1:**
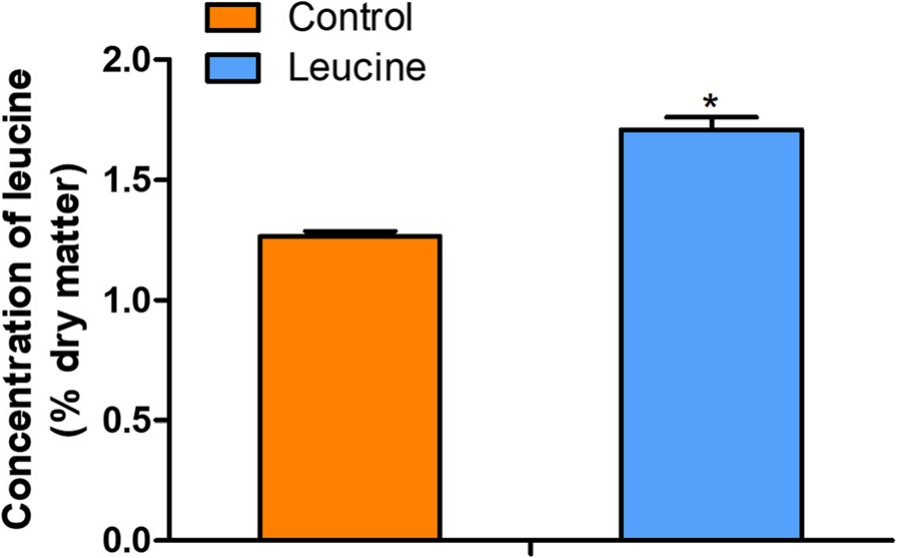
The leucine level in whole-body of zebrafish after early leucine programming at 13 dph. The value represented mean ± S. E.M. (*n* = 6), marked with an asterisk means significant level (*P* < 0.05)

We analyzed the leucine level in whole-body of zebrafish after early leucine programming at 13 dph. The result revealed that the leucine level in zebrafish of the leucine programming group was higher than that of the control group at 13 dph (*P* < 0.05) (Fig. 1). The total length of zebrafish in the leucine programming group was higher than that in the control group at 13 dph (*P <* 0.05). Meanwhile, the total length and body weight of zebrafish in the leucine programming group also increased significantly (*P <* 0.05) at 83 dph (Table 2). The content of total water, crude protein and crude lipid were detected in zebrafish larvas at 83 dph. The total water content showed no significant difference between the two groups (*P >* 0.05), and the content of total crude protein in the larvas of leucine programming group was significantly increased (*P <* 0.05), whereas the total crude fat was significantly decreased (*P <* 0.05) (Table 3).

**Table 2:** Growth performance of zebrafish at 13 and 83 dph

	13 dph		83 dph	
	Control	Leucine	Control	Leucine
Body weight (mg)	–	–	87.61 ± 3.10^a^	127.67 ± 2.84^b^
Total length (mm)	7.36 ± 0.25^a^	7.56 ± 0.22^b^	18.91 ± 0.35^a^	24.11 ± 0.22^b^

Data represent mean ± SEM (*n* = 6), values that share different letters are significantly different (*P* < 0.05)

**Table 3:** Body composition analysis of zebrafish at 83 dph

	Control	Leucine
Moisture (%)	74.14 ± 0.22	73.59 ± 0.29
Crude Protein (%)	16.03 ± 0.17^a^	18.27 ± 0.20^b^
Crude Lipid (%)	7.58 ± 0.09^a^	5.12 ± 0.10^b^

Data represents mean ± SEM (*n* = 6), values that share different letters are significantly different (*P* < 0.05)

### Effect of early leucine programming on mTOR signaling pathway

**Fig. 2:**
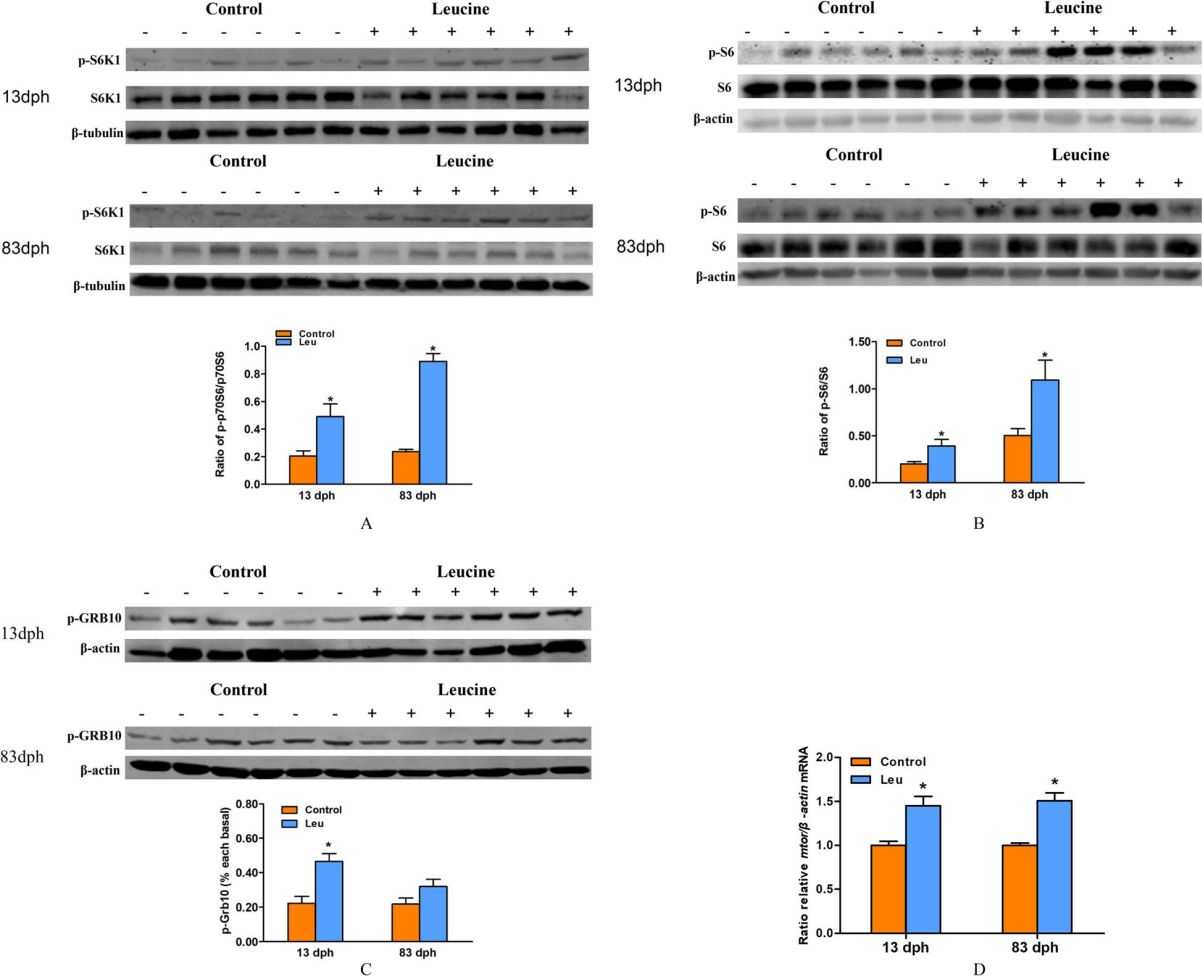
The phosphorylation status of mTOR downstream factors (S6K1, S6, Grb10) and the mRNA expression of *mtor* gene were displayed. Western blot analysis of phosphorylation status of mTOR downstream factors S6K1 (**a**), S6 (**b**) and Grb10 (**c**), meanwhile, the mRNA expression of *mtor* gene was analyzed by real-time qPCR (**d**). Each value is the mean ± S. E.M. (*n* = 6). Values marked with an asterisk means significant level (*P* < 0.05)

The phosphorylation status of mTOR downstream factors (S6K1, S6, Grb10) and the mRNA expression of *mtor* gene were displayed in Fig. 2. At 13 dph, the abundance of phosphorylated S6K1, S6 and Grb10 were increased in the larvas treated with leucine (*P* < 0.05). Furthermore, at 83 dph, compared with the control group, leucine programming also leaded to the higher abundance of phosphorylated S6K1 and S6 (*P* < 0.05). The abundance of phosphorylated Grb10 showed no significant difference between the two groups (*P* > 0.05) (Fig. 2a, b, c). Meanwhile, the mRNA expression of *mtor* gene was analyzed by real-time qPCR, and the mRNA levels of *mtor* in the leucine programming group were significantly higher than those in the control group at 13 dph and 83 dph (*P* < 0.05) (Fig. 2d).

### Expression of genes related to glucose, lipid and protein metabolism

**Fig. 3:**
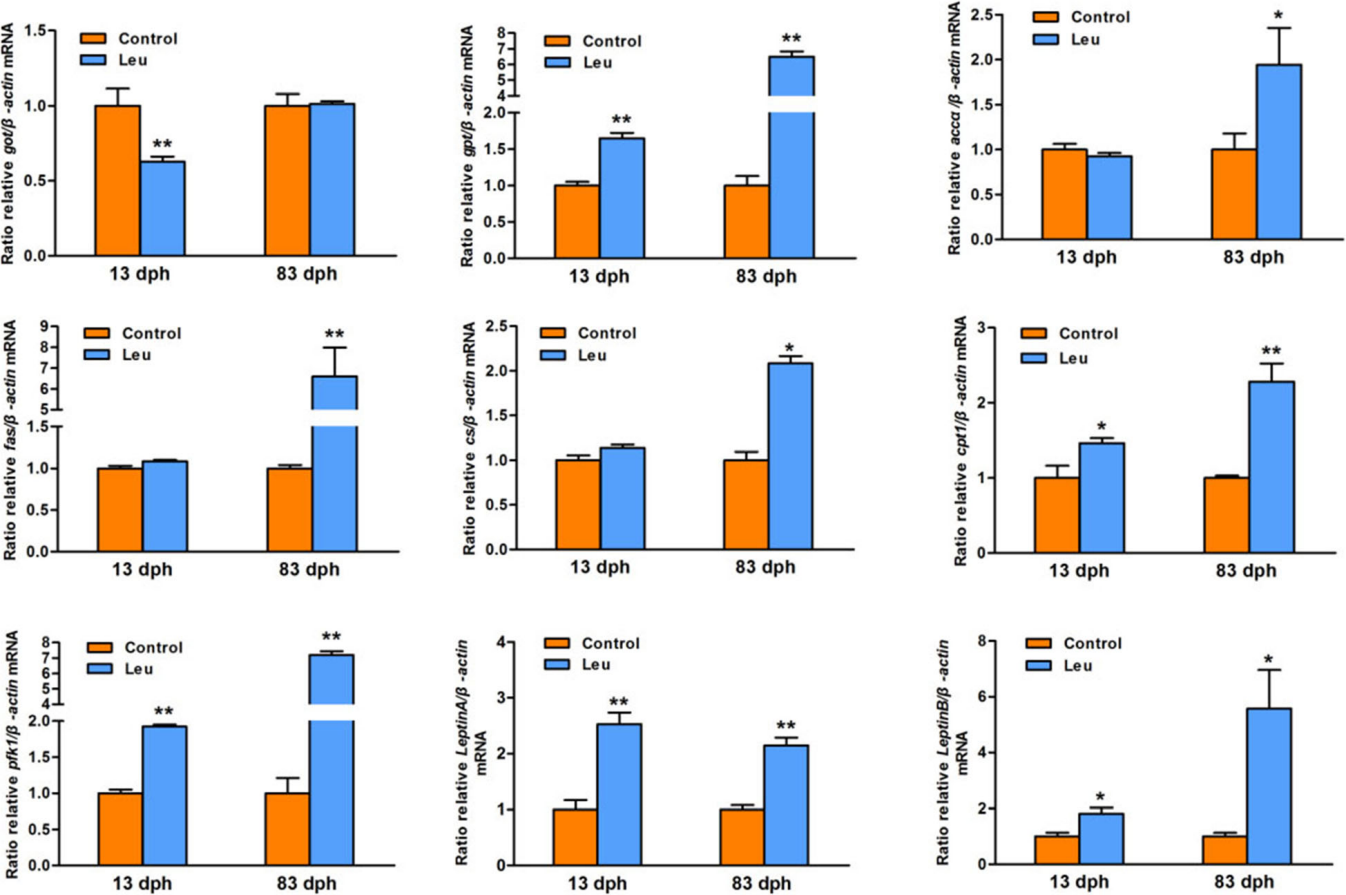
The mRNA expression of genes related to metabolism. The value represented mean ± S. E.M. (*n* = 6), marked with an asterisk means significant level (*P* < 0.05), marked with two asterisk means extremely significant level (*P* < 0.01)

The expression of genes involved in glucose, lipid and protein metabolism, were analyzed by real-time qPCR (Fig. 3). Compared with the control group, the mRNA levels of carnitine palmitoyl transferaseI (*cpt1*), phosphofructo kinaseI (*pfk1*), glutamic-pyruvic transaminase (*gpt*), and *leptin A* and *B* of zebrafish larvas in the leucine programming group were significantly higher at 13 dph and 83 dph (*P <* 0.05). The mRNA levels of citrate synthase (*cs*), acetyl-CoA carboxylase alpha (*accα*) and fatty acid synthase (*fas*) of fish in the leucine programming group were significantly increased at 83 dph (*P <* 0.01), but no significant difference at 13 dph. The abundance of glutamic-oxaloacetic transaminase (*got*) of fish in the leucine programming group was significantly decreased at 13 dph (*P <* 0.01), but no significant difference at 83 dph (*P >* 0.05).

### Bisulfite sequencing and DNA methylation profiling

To study the genome-wide DNA methylation pattern, we collected the liver tissues from zebrafish in the control group and leucine programming group for constructing genomic DNA libraries. Averagely 30 Gb clean bases after filtering low-quality reads, N reads and adaptor sequences were generated. The sequencing data in this study have been deposited in the Sequence Read Archive (SRA) database (accession number: SUB6149613 and PRJNA559591). The BS conversion rates of genomic DNA ranged from 99.44 to 99.51%. The high-quality methylation maps of the two groups were obtained, and the unique mapping rates ranged from 56.10 to 58.48% (Additional file 1). Proportion in total methyl-cytosine of mCG, mCHG and mCHH was summarized for each sample. In the control group, we detected 24,783,377 mC sites, 23,647,352 mCG cites (95.42% of all mC), 265,324 mCHG sites (1.07% of all mC), 870,701 mCHH sites (3.51% of all mC), respectively. Similarly, there were 95.57% mCG, 1.04% mCHG, and 3.39% mCHH in the leucine programming group (Additional file 2).

**Fig. 4:**
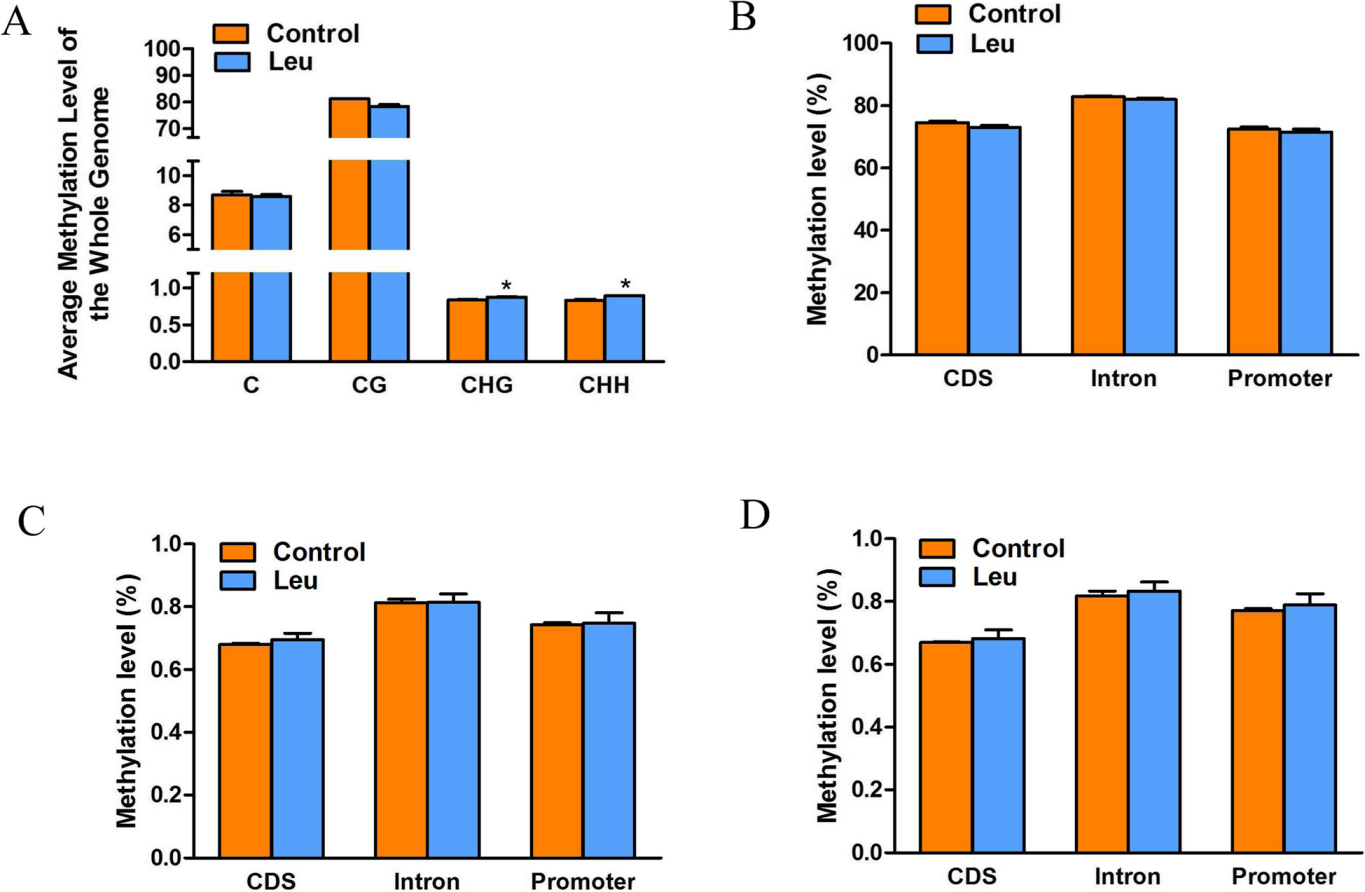
The average genome-wide methylation levels (**a**) and DNA methylation levels of different genomic regions (**b**-**d**). The value represented the mean ± S. E.M. (*n* = 3), marked with an asterisk means significant level (*P* < 0.05). A: X-axis represented methylation types, Y-axis represented average genome-wide methylation levels. B: X-axis represented genomics features, Y-axis represented methylation levels. **b** CG regions, **c** CHG regions, (**d**) CHH regions

We computed the average level of genome-wide methylation and found that the genome-wide methylation levels of total cytosine and CG methylation types showed no significant difference between the control and leucine programming group, while those of CHG and CHH methylation types in fish of the leucine programming group were significantly increased (Fig. 4a). To further study the global DNA methylation profile, we analyzed the DNA methylation levels of different genomic regions (Fig. 4b, c, d). The average methylation levels of different genomic regions showed no difference between the two groups. A major proportion of methylated sites were present in the regions of introns, and the average methylation level of CDSs was the lowest. In order to reveal the relationship between DNA methylation profiles and genes expression, we analyzed the DNA methylation profiles of transcriptional units which were divided into functional elements as shown in Additional file 3. Similar tendencies of methylation change were observed in different functional elements between the two groups.

DMRs were stretches of DNA in a sample’s genome that have different DNA methylation patterns compared with other samples, a sliding-window approach was used to identify DMRs which contained at least five CG (CHH or CHG) sites. We identified a total of 199,750, 671 and 8314 DMRs in CG, CHG, and CHH contexts (CHH-DMRs, CHG-DMRs, and CG-DMRs) between zebrafish of the control group and leucine programming group at 83 dph, respectively. The CG-DMRs, CHG-DMRs and CHH-DMRs were located in 44,459, 1128 and 9572 genes, respectively. The vast majority of DMRs (95.7%) were in the CG context, while only 4.3% DMRs were in CHG and CHH.

DMRs-related genes were analyzed using KEGG database. In gene body region, 304, 241 and 297 pathways were identified from CG-DMRs, CHG-DMRs and CHH-DMRs, respectively. Furthermore, in promoter region, 302, 75 and 226 pathways were identified from CG-DMRs, CHG-DMRs and CHH-DMRs, respectively. The top 20 pathways in ascending order of corrected *p* value were listed in Additional file 4. To investigate pathways and processes that may be subject to epigenetic variation in association with DMRs, we conducted a GO enrichment analysis. It is revealed that genes involved in GO terms such as cellular process, biological regulation, metabolic process, binding, catalytic activity, and response to stimulus were significantly over-represented. The top 60 GO terms were listed in Additional file 5 by ascending order of corrected p value.

### Validation of target DMGs by real-time qPCR

**Fig. 5:**
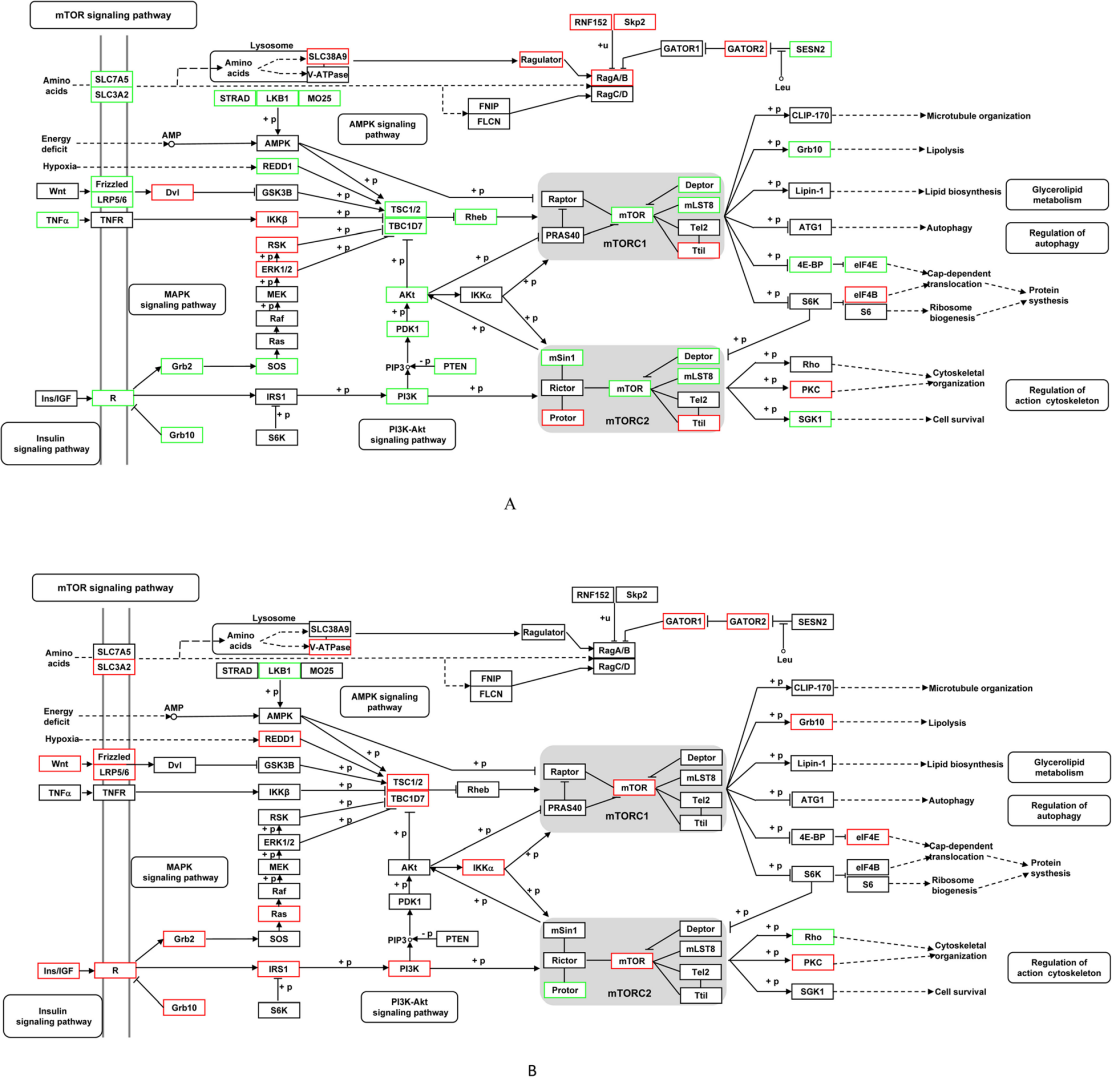
Differentially methylated genes of mTOR signaling pathway at CG (**a**) and CHH types (**b**). Compared with the control group, the red marked that the methylation levels of the genes were higher in zebrafish of the leucine programming group, and the green marked the lower levels

The present study focused on the mTOR signaling pathway. According to the bisulfite sequencing, we found that 28 CG-DMGs (such as *mTOR*, *Deptor*, *eIF4E*, *4E-BP*, *Grb10*, *mLST8* and *SGK1*) involved in mTOR signaling pathway, exhibited the lower levels of DNA methylation in fish of the leucine programming group than those of the control group (Fig. 5a). Meanwhile, we also found that 21 CHH-DMGs (such as *mTOR*, *Grb10*, *GATOR1*, *GATOR2* and *eIF4E*) involved in mTOR signaling pathway, exhibited the higher levels of DNA methylation in fish of the leucine programming group than those of the control group (Fig. 5b).

**Fig. 6:**
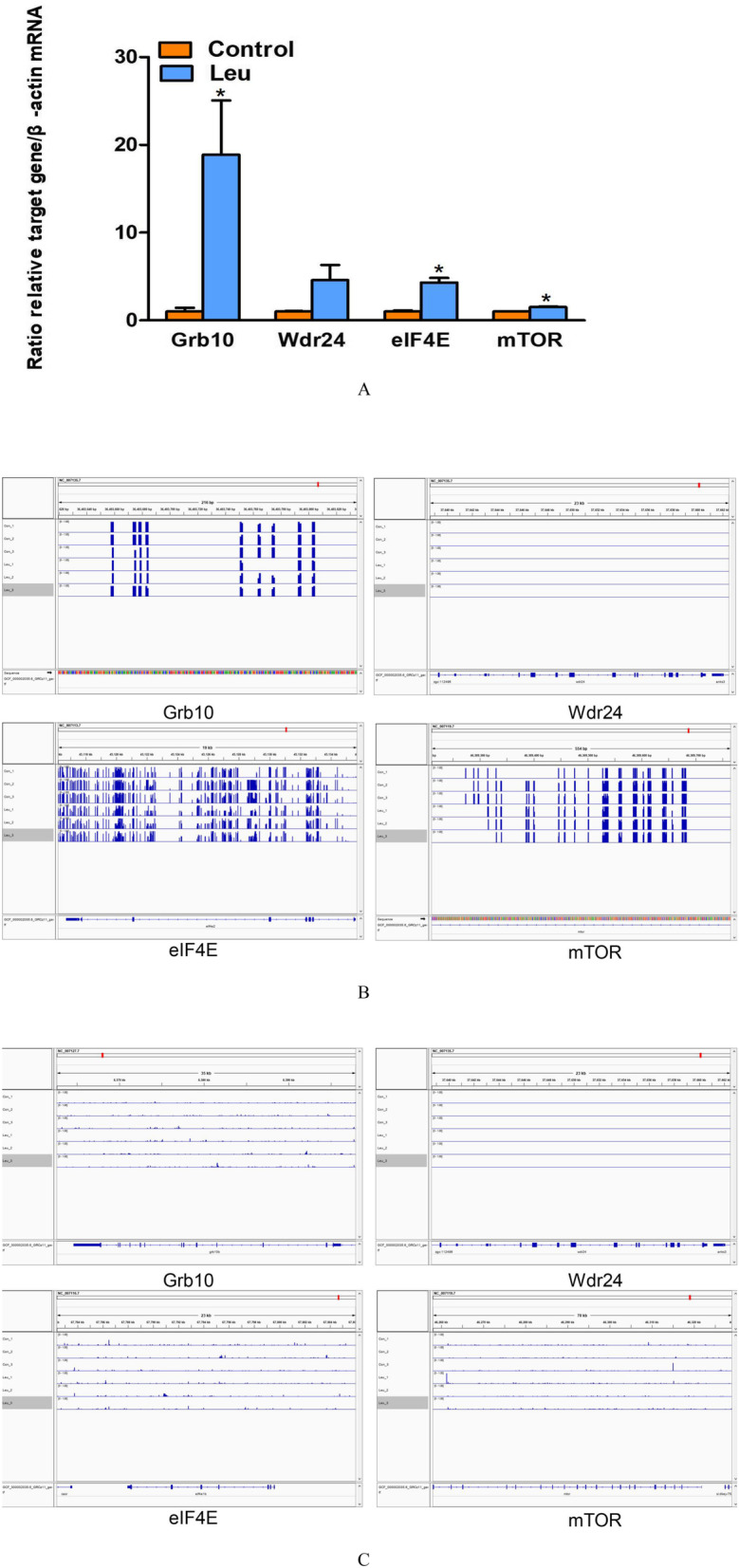
The mRNA expression (**a**), the methylation status in CG (**b**) and CHH (**c**) type of DMGs involved in mTOR signaling pathway. All qRT-PCR reactions were performed with six biological replicates, the value represented the mean ± S. E.M. marked with an asterisk means significant level (*P* < 0.05). Displayed DNA methylation status of *Grb10*, *eIF4E*, *mTOR* and *Wdr24* by IGV tool. Con-1, Con-2, Con-3: samples of the control group; Leu-1, Leu-2, Leu-3: samples of the leucine programming group

The changes of methylation levels in genomic regions could be associated with the differential expression of genes. To analyze the expression of the DMGs in mTOR signaling pathway, the real-time qPCR was carried out for four DMGs in fish from the leucine programming group and control group. As shown in Fig. 6a, the mRNA levels of *Grb10*, *eIF4E* and *mTOR* genes were significantly higher in fish of the leucine programming group than those of the control group (*P <* 0.05). Meanwhile, the methylation status of *Grb10*, *eIF4E*, *mTOR* and *Wdr24* genes in CG and CHH types were shown in Fig. 6b and c.

## Discussion

Several studies in mammals reported that nutrient and metabolic programming during the critical periods of early development might result in the long-term genetic and physiological consequences during adulthood [1, 29–31]. The concept of early nutritional programming has been applied to improve the nutrient utilization in adult fish [3, 4, 6]. However, no effect is recorded for nutritional programming of amino acids. In the present study, we attempted to treat zebrafish larvas with leucine at early developmental stage for assessing the short-term and long-term modifications of early leucine programming on growth and nutritional utilization.

Leucine participates in the regulation of protein metabolism through mTOR signaling pathway [32, 33]. However, it has not yet been determined if leucine could improve the growth and nutritional utilization by early stimulation. The leucine level in whole-body of zebrafish after early leucine programming was significantly higher than that of the control group at 13 dph, suggesting that the treatment of 1% leucine could change the leucine content of zebrafish. Zebrafish larvas were treated with leucine from 3 dph to 13 dph, and the growth was significantly better than those in the control group at 13 and 83 dph. Previous study reported that the growth and survival rate were affected by neither early glucose stimulus nor dietary challenge with high carbohydrates [34]. Early hyperglucidic stimulation had no significant effect on body weight in rainbow trout and zebrafish during a long experimental period [3, 6]. However, an early stimulus of high carbohydrate diet (60%) at first-feeding can lead to the detrimental effects in the long-term growth performance [35]. In the present study, the early leucine programming could markedly promote the growth of zebrafish at both early developmental stage and adult stage. By the body composition analysis at 83 dph, we found that the crude protein content of zebrafish with early leucine programming was increased, while the crude lipid was decreased, and the total water showed no difference. The study in gilthead seabream (*Sparus aurata*) juvenile, showed that early glucose stimuli at the larval stage has the positive effect on lipid retention, but not on protein saving [36]. However, it is suggested that early leucine programming could effectively improve the growth of zebrafish, which might be attributed to the increased protein synthesis and lipid decomposition.

Leucine activates the utilization of amino acids for protein synthesis and inhibits protein catabolism through mTOR pathway [12, 37]. To investigate whether early leucine programming in zebrafish can promote the growth and protein synthesis through activating mTOR pathway. We examined the phosphorylation status of mTOR downstream factors S6K1, S6 and Grb10, and found that the early leucine programming could continuously activate the mTOR signaling pathway at both 13 and 83 dph, with higher phosphorylation status of S6K1 and S6. In addition, the mRNA level of *mtor* gene was also increased in the leucine programming group, suggesting the activation of mTOR signaling. The leucine in the diet plays an important role in regulating growth performance, body composition, and mTOR signaling pathways in fish [14, 38, 39]. With the injection of leucine in the hypothalamus of rainbow trout, the mTOR signaling pathway in central nervous system is activated, thereby regulating the digestion and absorption of nutrients [15]. In rainbow trout hepatocytes, amino acids up-regulate the protein synthesis by activating mTOR signaling pathway [16, 17]. We therefore surmised that the early leucine programming could effectively activate the mTOR signaling pathway, which might promote protein synthesis in a long-term period of zebrafish.

In juvenile blunt snout bream (*Megalobrama amblycephala*), dietary leucine supplement can affect glucose metabolism and lipogenesis involved in mTOR signaling pathway [40]. To study the effect of early leucine stimulation on nutritional metabolism, we analyzed the mRNA expression of genes related to nutritional metabolism in zebrafish. GOT and GPT are the important amino acid-degrading enzymes, and their activities positively correlated to dietary protein levels in Jian carp (*Cyprinus carpio var. Jian*) [41]. With the early leucine programming, the *gpt* mRNA expression was upregulated in the zebrafish at 83 dph, whereas the *got* was not significantly affected. The gene expressions of lipogenesis enzymes (*accα, fas* and *cs*) were increased with the early leucine programming, and the gene expression of fat β-oxidation key enzyme (*cpt1*) was also significantly increased, suggesting that the early leucine programming could accelerate the synthesis and oxidative decomposition of lipid, improving the utilization of lipid. In rainbow trout hepatocytes, leucine could activate the mTOR signaling pathway to up-regulate the gene expression of *fas*, promoting lipid synthesis [16]. In addition, for glucose metabolism, the mRNA level of glycolysis gene (*pfk1*) was also significantly elevated by the early leucine programming. We also found a significant up-regulation of *leptin A* and *B* in the leucine programming group. Leptin signaling regulates glucose homeostasis but is not an adipostatic factor in zebrafish [42]. mTORC1 is sufficient to affect metabolic pathways by activation of a transcriptional program for metabolic gene targets of sterol regulatory element-binding protein (SREBP), including glycolysis, pentose phosphate pathway and lipid biosynthesis [43]. Therefore, early leucine stimulation could increase the phosphorylation level of protein S6K1 and S6, as the regulatory signaling of mTOR pathway, promoting protein synthesis and growth. Meanwhile, the glucose and lipid metabolism were also significantly enhanced, which might be related to mTOR signaling, in zebrafish with early leucine programming.

There are increasing evidences that the early environmental stimulation might cause changes in organisms via epigenetic modification. Previous study in Nile tilapia (*Oreochromis niloticus*) gonads has observed the DNA methylation changes on a genome-wide scale after earlier high-temperature induction [44]. Zebrafish embryos are exposed to androgens (testosterone and dihydrotestosterone) early at 26 to 56 h post fertilization, resulting in transgenerational alterations in the zebrafish ovarian epigenome [45]. In the present study, early leucine stimulation led to the long-term changes of gene expression in zebrafish. We compared the genome-wide methylation patterns between the control and leucine programming zebrafish. The methylation level of CpGs in zebrafish liver was above 70%, similar to those found in zebrafish and tilapia [46, 47]. The total DNA methylation level of CHG and CHH methylation types in fish of the leucine programming group was higher than those of the control group. In addition, we identified the pathways associated with energy metabolism in early leucine programming. Among these pathways, the mTOR signaling pathway is an attractive target, because it plays an important role in the integration between amino acid and energy-sensing pathways [48]. The most genes of mTOR pathway were hypomethylated (lower methylation) for CG type and hypermethylated (higher methylation) for CHH type in the leucine stimulation group compared with the control group. These results suggested that the patterns of DNA methylation could be highly plastic and react to the cues of early nutrition induction.

To highlight the potential role of methylation of key genes in mTOR signaling pathway with early leucine programming, we examined the mRNA expression of four genes in mTOR signaling pathway, including *Grb10*, *eIF4E*, *mTOR* and *Wdr24*. The result showed the increased mRNA expression of *Grb10*, *eIF4E* and *mTOR* genes in the zebrafish with early leucine programming. *Grb10* is a key regulator of the mTORC1 signaling pathways on lipid metabolism [49]. The enhanced phosphorylation state of eukaryotic initiation factor 4E-binding protein 1 (4E-BP1) induced by administration of leucine stimulates protein synthesis by accelerating translation initiation [50]. We suggested that the decreased methylation in CG type and increased methylation in CHH type of *Grb10*, *eIF4E* and *mTOR* genes, could contribute to their enhanced gene expressions. There are complex relationships between DNA methylation and gene expression. Generally, DNA methylation in promoters is negatively associated with gene expression. In Nile tilapia, high temperature increases the DNA methylation level and decreases the mRNA expression of *cyp19a1a* gene [51]. However, studies have also shown that DNA methylation is not necessarily associated with the repression of gene expression, but exhibits the positive correlations with transcription activation instead [52]. In the study of *Brassica napus*, the gene expression of BnaA0724700D and BnaA08g08410D are up-regulated, although they exhibit opposite methylation patterns in their promoters [53]. In the present study, the increased methylation in CHH type were at the gene body region of *Grb10*, *eIF4E* and *mTOR* genes. Therefore, we speculated that the enhanced expression of *Grb10*, *eIF4E* and *mTOR* genes in the zebrafish with early leucine programming might be more possibly attributed to the decreased methylation in CG type at the gene promotor regions. The study of genome-wide methylome has emphasized that the promoter methylation is closely related to gene regulation [54].

## Conclusions

For the first time, we used the DNA methylation profiling to elucidate the regulatory mechanism of early amino acid programming on nutritional utilization. We found that the DNA methylation of genes involved in mTOR signaling pathway may contribute to the activation of mTOR signaling, promoting protein synthesis and growth of zebrafish with early leucine programming. In addition, early leucine programming could enhance the mRNA expressions of genes related to glycolipid metabolism. The present study may be beneficial for better understanding the epigenetic regulation in nutrition metabolism by early programming.

## Supplementary information

**Additional file 1.** Sequencing data by whole genome bisulfite sequencing (WGBS).

**Additional file 2.** Comparison of DNA methylation patterns between the two groups.

**Additional file 3.** DNA methylation levels across genomic elements. The abscissa represented different functional elements that a, b, c, d, e, f and g denoted upstream, first exon, first intron, inner exon, inner intron, last exon and downstream, respectively. The left ordinate represented the mean methylation levels of CG, and the right ordinate represented the mean methylation levels of CHG/CHH. The dotted, green, vertical line represented the TSS, and the red, orange and blue solid lines represented CG, CHH and CHG, respectively, which showed the methylation levels fluctuating in the different regions.

**Additional file 4 **Scatterplot of enriched KEGG pathways for the differentially methylated genes in promoter and gene body regions. The ordinate represented the enriched pathways, and the abscissa represented the rich factor of corresponding pathways; the size of the spots represented the number of genes related to DMRs enriched in each pathway, while the color of the spot represented the corrected *p* value of each pathway.

**Additional file 5.** Gene ontology functional annotations for the differentially methylated genes. All GO terms were divided into three categories: blue refer to biological process, green refer to cell components, and red refer to molecular function. The ordinate represents three domains of GO while abscissa represents the gene number in every pathway and processes.

## Data Availability

The sequencing data in this study have been deposited in the Sequence Read Archive (SRA) database (accession number: SUB6149613 and PRJNA559591). The datasets used and/or analyzed during the current study are available from the corresponding author on reasonable request.

## Abbreviations

dph: Days post hatching
Grb10: Growth factor receptor-bound protein 10
eIF4E: Eukaryotic translation initiation factor 4E
mTOR: Mechanistic target of rapamycin
S6: Ribosomal protein S6
S6K1: Ribosomal protein S6 kinase 1
WGBS: Whole genome bisulfite sequencing
DMRs: Differentially methylated regions
IGV: Integrative Genomics Viewer
cpt1: Carnitine palmitoyl transferase I
pfk1: Phosphofructo kinase I
gpt: Glutamic-pyruvic transaminase
cs: Citrate synthase
accα: Acetyl-CoA carboxylase alpha
fas: Fatty acid synthase
Wdr24: WD repeat-containing protein 24
SREBP: Sterol regulatory element-binding protein
4E-BP1: 4E-binding protein 1
BSMAP: Bisulfite Sequence Mapping Program
KEGG: Kyoto Encyclopedia of Genes and Genomes
SRA: Sequence Read Archive
