# Supplementary material for: Early leucine programming on protein utilization and mTOR signaling by DNA methylation in zebrafish (Danio rerio)
Source: Nutr Metab (Lond). 2020 Aug 14;17:67. doi: 10.1186/s12986-020-00487-3 (PMC7427859; doi:10.1186/s12986-020-00487-3)
Supplement: Supplementary file 1 — Additional file 1. Sequencing data by whole genome bisulfite sequencing (WGBS). [file 12986_2020_487_MOESM1_ESM.docx]

**Additional file 1. Sequencing data by whole genome bisulfite sequencing (WGBS).**

| Sample ID | Clean Reads | Mapped Reads | Mapping Rate (%) | Uniquely Mapped Reads | Uniquely Mapping Rate (%) | Bisulfite Conversion Rate (%) |
| --- | --- | --- | --- | --- | --- | --- |
| Con-1 | 200,000,002 | 144,947,611 | 72.47 | 115,503,706 | 57.75 | 99.46 |
| Con-2 | 200,000,004 | 146,955,953 | 73.48 | 116,066,455 | 58.03 | 99.46 |
| Con-3 | 200,000,002 | 142,292,578 | 71.15 | 112,199,230 | 56.1 | 99.47 |
| Leu-1 | 200,000,004 | 148,208,598 | 74.1 | 116,953,052 | 58.48 | 99.51 |
| Leu-2 | 200,000,002 | 145,392,589 | 72.7 | 114,729,624 | 57.36 | 99.46 |
| Leu-3 | 200,000,002 | 145,364,783 | 72.68 | 114,376,500 | 57.19 | 99.44 |

Note: Con-1, Con-2, Con-3: samples of the control group; Leu-1, Leu-2, Leu-3: samples of the leucine programming group.
