# Supplementary material for: Early leucine programming on protein utilization and mTOR signaling by DNA methylation in zebrafish (Danio rerio)
Source: Nutr Metab (Lond). 2020 Aug 14;17:67. doi: 10.1186/s12986-020-00487-3 (PMC7427859; doi:10.1186/s12986-020-00487-3)
Supplement: Supplementary file 2 — Additional file 2. Comparison of DNA methylation patterns between the two groups. [file 12986_2020_487_MOESM2_ESM.docx]

**Additional file 2. Comparison of DNA methylation patterns between the two groups.**

| Sample ID |  | mCG | mCHG | mCHH |
| --- | --- | --- | --- | --- |
| Con-1 | mC number | 23,379,213 | 264,489 | 877,517 |
|  | Proportion (%) | 95.343 | 1.079 | 3.579 |
| Con-2 | mC number | 23,699,799 | 262,650 | 849,731 |
|  | Proportion (%) | 95.517 | 1.059 | 3.425 |
| Con-3 | mC number | 23,863,044 | 268,833 | 884,854 |
|  | Proportion (%) | 95.388 | 1.075 | 3.537 |
| Leu-1 | mC number | 24,367,012 | 254,314 | 823,001 |
|  | Proportion (%) | 95.766 | 0.999 | 3.235 |
| Leu-2 | mC number | 23,888,455 | 259,582 | 856,830 |
|  | Proportion (%) | 95.535 | 1.038 | 3.427 |
| Leu-3 | mC number | 23,750,836 | 269,049 | 873,432 |
|  | Proportion (%) | 95.41 | 1.081 | 3.509 |

Note: Con-1, Con-2, Con-3: samples of the control group; Leu-1, Leu-2, Leu-3: samples of the leucine programming group. mCG: numbers of methylated cytosine in CG context and percentage of methylated cytosine in whole genome; mCHG: numbers of methylated cytosine in CHG context and percentage of methylated cytosine in whole genome; mCHH: numbers of methylatedcytosine in CHH context and percentage of methylated cytosine in whole genome.
