# Supplementary figures and images for: Early leucine programming on protein utilization and mTOR signaling by DNA methylation in zebrafish (Danio rerio)
Source: Nutr Metab (Lond). 2020 Aug 14;17:67. doi: 10.1186/s12986-020-00487-3 (PMC7427859; doi:10.1186/s12986-020-00487-3)

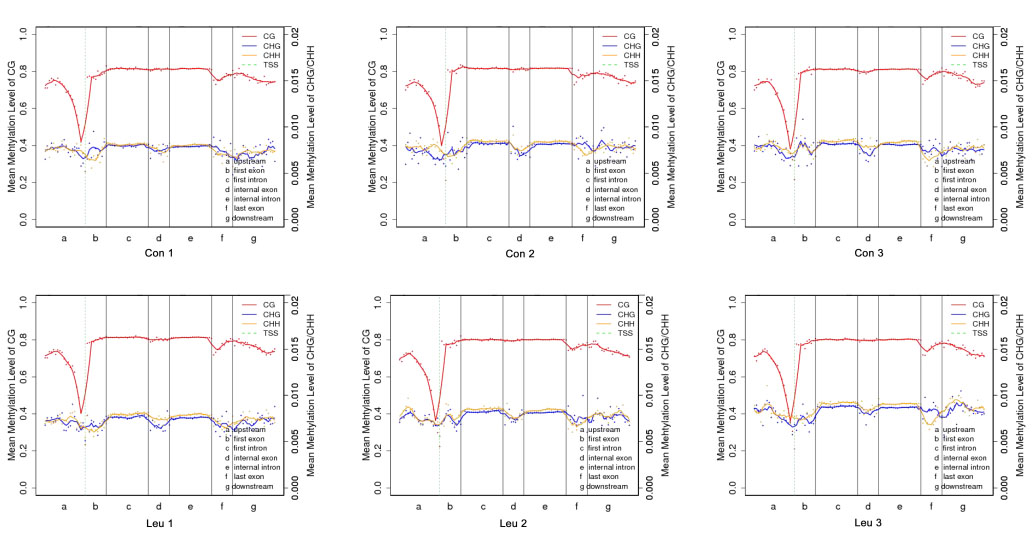

Supplement: Supplementary file 3 — Additional file 3. DNA methylation levels across genomic elements. The abscissa represented different functional elements that a, b, c, d, e, f and g denoted upstream, first exon, first intron, inner exon, inner intron, last exon and downstream, respectively. The left ordinate represented the mean methylation levels of CG, and the right ordinate represented the mean methylation levels of CHG/CHH. The dotted, green, vertical line represented the TSS, and the red, orange and blue solid lines represented CG, CHH and CHG, respectively, which showed the methylation levels fluctuating in the different regions. [file 12986_2020_487_MOESM3_ESM.jpg]

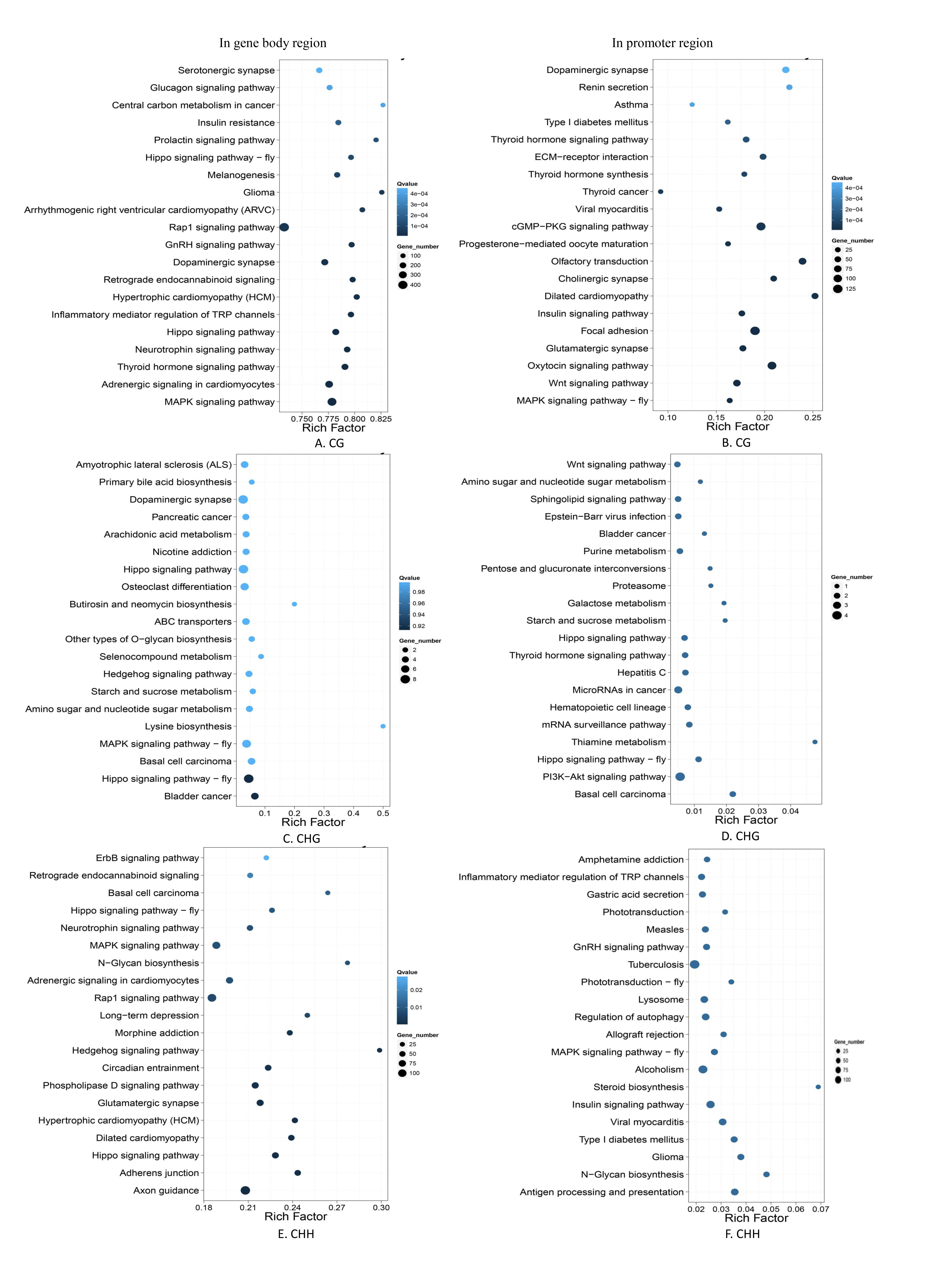

Supplement: Supplementary file 4 — Additional file 4 Scatterplot of enriched KEGG pathways for the differentially methylated genes in promoter and gene body regions. The ordinate represented the enriched pathways, and the abscissa represented the rich factor of corresponding pathways; the size of the spots represented the number of genes related to DMRs enriched in each pathway, while the color of the spot represented the corrected p value of each pathway. [file 12986_2020_487_MOESM4_ESM.jpg]

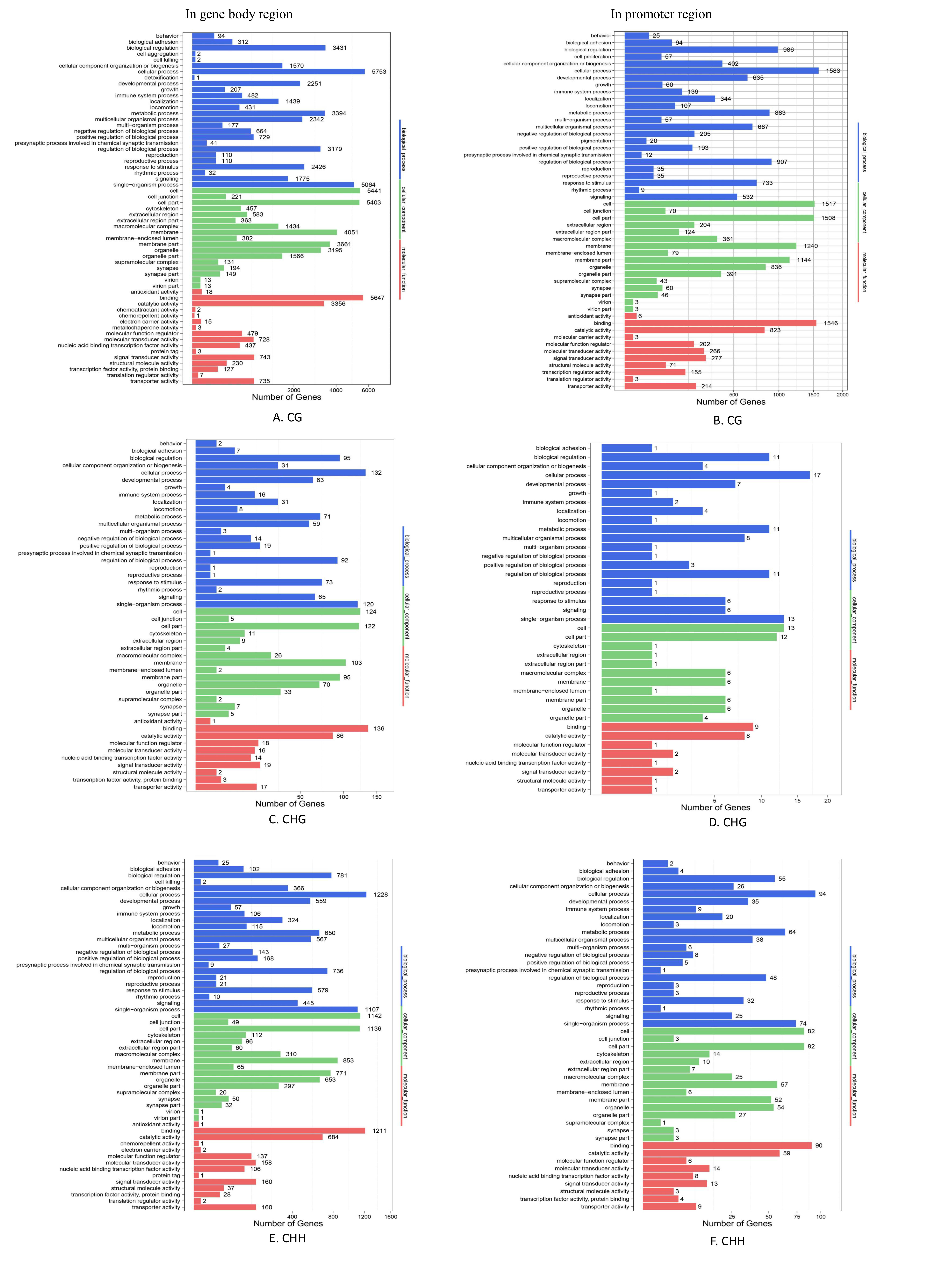

Supplement: Supplementary file 5 — Additional file 5. Gene ontology functional annotations for the differentially methylated genes. All GO terms were divided into three categories: blue refer to biological process, green refer to cell components, and red refer to molecular function. The ordinate represents three domains of GO while abscissa represents the gene number in every pathway and processes. [file 12986_2020_487_MOESM5_ESM.jpg]
